# The Effect of Antihypertensive Drugs on NADH in Newly Diagnosed Primary Hypertension

**DOI:** 10.1155/2022/6159883

**Published:** 2022-03-31

**Authors:** Regina Pawlak-Chomicka, Tomasz Krauze, Pawel Uruski, Jaroslaw Piskorski, Andrzej Wykretowicz, Andrzej Tykarski, Przemyslaw Guzik

**Affiliations:** ^1^Department of Hypertensiology, Angiology and Internal Medicine, Poznan University of Medical Sciences, Poznan, Poland; ^2^Department of Cardiology-Intensive Therapy and Internal Medicine, Poznan University of Medical Sciences, Poznan, Poland; ^3^Institute of Physics, University of Zielona Gora, Zielona Gora, Poland

## Abstract

**Background:**

Some antihypertensive medications alter cellular energy production, presumably by modification of the mitochondrial function. In vivo studies of such effects are challenging in humans. We applied a noninvasive forearm skin measurement of the 460-nm fluorescence specific for the reduced form of nicotinamide adenine dinucleotide (NADH) to study the 6-week effects of four different antihypertensive medications on mitochondrial function using the Flow-Mediated Skin Fluorescence (FMSF).

**Methods:**

In a prospective open-label study, we compared the long-term effects of a 6-week treatment with either amlodipine (5 mg), perindopril (5 mg), nebivolol (5 mg), or metoprolol (50 mg) on the dynamic flow-mediated changes in the skin NADH content in 76 patients (29 women) with untreated primary arterial hypertension (HA). Patients underwent 24-hour ambulatory blood pressure monitoring. To study mitochondrial function, the FMSF was measured at rest, during 100-second ischemia and postischemic reperfusion. The control group consisted of 18 healthy people (7 women).

**Results:**

There were no significant differences in the FMSF parameters between the control and the study group before medication. After the 6-week treatment, all drugs similarly reduced blood pressure. Neither amlodipine, perindopril, nor nebivolol changed the flow-mediated 460-nm skin fluorescence significantly. However, metoprolol raised this fluorescence at rest, during ischemia and reperfusion (*P* at most <0.05), indicating an increase in the total NADH skin content.

**Conclusion:**

Amlodipine, perindopril, and nebivolol appear neutral for the skin NADH content during the 6-week antihypertensive treatment. Similar treatment with metoprolol increased skin NADH at rest, during ischemia and reperfusion, probably due to an effect on microcirculation and altered mitochondrial function. Explanation of the potential mechanisms behind metoprolol influence on the skin NADH metabolism requires further investigation.

## 1. Introduction

The concentration of the reduced form of nicotinamide adenine dinucleotide (NADH) varies depending on the condition and prosperity of cells. The nucleotide's reduced form is not converted to the oxidized form of nicotinamide adenine dinucleotide (NAD^+^) and accumulates without oxygen. It happens during hypoxia, anoxia, and cell death [1]. The NADH amount increases in cardiovascular diseases [2] and with age [3].

NADH originates in the mitochondria, but its trace amount is also found in cytoplasm [1]. It plays a crucial role in cell metabolism by driving electron transport chains and modulating the last stage of cellular respiration under aerobic conditions [4] to produce ATP (adenosine triphosphate). Therefore, the amount of NADH is an indicator of mitochondrial function [1].

NADH concentration has been assessed in many diseases, e.g., coronary heart disease, chronic obstructive pulmonary disease, and diabetes [3, 5, 6]. In patients with coronary heart disease, NADH is associated with some plasma endothelial markers, such as levels of asymmetric dimethylarginine and endothelin-1 [5]. In diabetes, NADH helps in detecting microcirculation pathologies and metabolic regulation [3]. Majewski et al. showed that NADH might identify an impaired microcirculation in patients with chronic obstructive pulmonary disease [6]. However, no studies have assessed the role of NADH in arterial hypertension, which is an essential factor increasing cardiovascular risk [7], e.g., by deteriorating the function of the endothelium [8], and thus damage to the microcirculation [9]. NADH measurement in the skin has appeared to be helpful in the general modeling of microcirculation function [2, 10].

Flow-Mediated Skin Fluorescence (FMSF) is a noninvasive method measuring skin fluorescence proportional to NADH [11]. The FMSF measures the 460 nanometers (nm) skin fluorescence of NADH at rest, during transient ischemia and reperfusion, giving an indirect insight into the function of microcirculation and mitochondria [2, 12–14]. This study aimed to estimate the effect of hypotensive drugs on skin NADH measured by the FMSF in newly diagnosed primary hypertension.

## 2. Materials and Methods

This study involved 76 patients with newly diagnosed primary hypertension, based on 24-hour ambulatory blood pressure monitoring (ABPM), according to the European Society of Hypertension 2018 Guidelines [15] and 18 healthy volunteers as a control.

Inclusion criteria were primary hypertension not exceeding 180/110 mmHg and sinus rhythm on electrocardiogram. Exclusion criteria were secondary hypertension, other chronic diseases, including diabetes, chronic obstructive pulmonary disease, cancer, other cardiovascular diseases, autoimmune diseases, renal impairment (glomerular filtration rate <60 ml/min/1.73 m^2^), acute inflammation, pregnancy, or breastfeeding.

Patients were divided into four therapeutic groups, randomly assigned to one of the following drugs: amlodipine, perindopril, nebivolol, and metoprolol. Each patient was admitted for two visits before starting treatment (v1) and after six weeks of antihypertensive therapy (v2).

**Figure 1:**
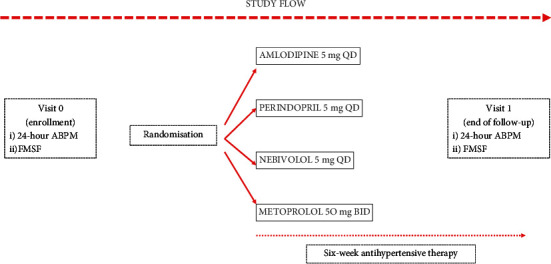
The study protocol. Patients with untreated hypertension were randomised to one of the open-label antihypertensive treatment modes and were prescribed either amlodipine 5 mg once a day (QD) or perindopril 5 mg QD, or nebivolol 5 mg QD or metoprolol 50 mg twice a day (BID). Measurements of the 24-hour ambulatory blood pressure monitoring (ABPM) and the flow-mediated 460 nm skin fluorescence (FMSF) were made at visit 1 before starting the prescribed treatment and at visit 2 after the six-week therapy. Patients were asked to take their morning dose of the prescribed medication on the day of visit 2.

ABPM and Flow-Mediated Skin Fluorescence (FMSF) measurements were performed at each visit. The simplified protocol is presented in Figure 1.

### 2.1. Flow-Mediated Skin Fluorescence

The method involves a measurement of the intensity of the light emitted by NADH (in the 420–480 nm range) created by initial excitation of particles with an absorption wave (320–380 nm), reaching about 0.3–0.5 mm deep into the skin [5, 16]. The measurement is taken on the skin of the forearm at rest, then during short-term controlled ischemia due to complete closure of the brachial artery by the blood pressure cuff and during reperfusion. The research method was described by Bugaj et al. [17]. The following parameters were selected in the description of the fluorescence signal changes:

- Bmean (baseline mean) (kFU): mean fluorescence recorded before ischemia as the baseline value.
- FImax (fluorescence ischemia maximum) (kFU): the maximal fluorescence above the baseline during ischemia.
- FRmin (fluorescence reperfusion minimum) (kFU): the minimal fluorescence below the baseline during reperfusion.
- Imax (ischemia maximum) (kFU): the difference between FImax and Bmean.
- Rmin (reperfusion minimum) (kFU): the difference between Bmean and FRmin.
- IRampl (ischemia reperfusion amplitude) (kFU): the difference between FImax and FRmin.
- CImax (contribution ischemia maximum): Imax/IRampl ratio.

**Figure 2:**
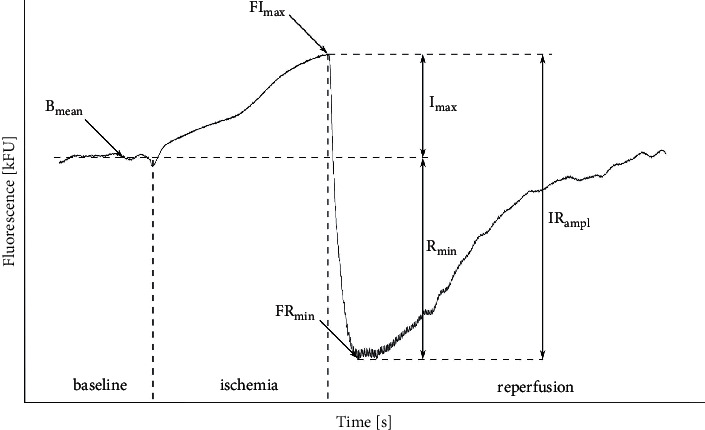
This is an example of the 460 nm skin fluorescence recorded at rest, during 100-second transient ischemia and the following reperfusion. For details on measured parameters, refer to the main text in the methodology section.

The baseline condition of the tissue describes Bmean. Other parameters represent the response to temporary ischemia, thus incorporating FMSF into the microcirculation assessment methods [3]. An example of the fluorescence signal path and the determination of the measured parameters are presented in Figure 2.

### 2.2. Statistical Analysis

All data were expressed as medians (lower and upper quartiles, Q1 and Q3) or mean (standard deviation, SD) of the subjects. The participants were divided into four groups according to the drug used. The statistical analysis was performed using the STATISTICA program. The Shapiro–Wilk test was used to check the normality of the decomposition. The Wilcoxon test was performed to present the ABPM and FMSF parameters. The ANOVA or Kruskal–Wallis test was employed to check differences between groups in terms of age, BMI (body mass index), ABPM parameters, pulse rate (PR), and FMSF parameters during v1 and v2. The Mann–Whitney test was used to compare the FMSF parameters of the control group and the study group at v1. Differences with a probability value of <0.05 were considered statistically significant.

To compare the effects of various antihypertensive medications on the FMSF before and after the treatment, we used the paired *t*-test for the Bmean, which corresponds to the reference or resting value of the 460 nm skin fluorescence, and usually, its results present normal distribution. With the additional assumptions of the alpha value set at 0.05 and the beta value for the statistical power set at 0.8, the estimated minimum sample size was 14 subjects for each antihypertensive drug group.

## 3. Results

The study group consisted of 76 participants divided into four groups depending on the drug used. The control group consisted of 18 healthy volunteers (7 women, 39% of the group) with an average age of 37.9 (±11.0). The characteristics of the study group are presented in Table 1. The groups of patients did not differ in terms of age, BMI, systolic and diastolic blood pressure in both day and night measurements and FMSF parameters measured at v1. There were also no differences in FMSF parameters between the control and study groups during v1.

### 3.1. Blood Pressure and Heart Rate

In all subgroups, blood pressure was significantly reduced by the applied therapy (Tables 2–5). The most substantial decrease in both systolic and diastolic pressure was achieved in the metoprolol group. The weakest hypotensive effect was obtained in the group using amlodipine. However, the difference in reducing office blood pressure between the groups was not significant. Changes in heart rate values measured at both visits, according to drug subgroups, are presented in Tables 2–5. The pulse value significantly changed when using nebivolol and metoprolol. The ANOVA test showed no significant difference in heart rate decrease in the nebivolol versus metoprolol group.

### 3.2. NADH

Changes in NADH level between two visits varied depending on the treatment used.

### 3.3. Amlodipine (Table 2)

In the amlodipine group, values of basal line, maximal fluorescence, and minimal fluorescence during reperfusion on v2 decreased compared to v1. However, these changes were not statistically significant.

### 3.4. Perindopril (Table 3)

For the perindopril group, the signal path also did not change significantly. The changes consisted of elevation of the basal line and an increase in the maximal fluorescence signal in ischemia. However, the minimal fluorescence in the reperfusion phase was similar to that on the first visit.

### 3.5. Nebivolol (Table 4)

The basal line fluorescence signal was observed in the nebivolol group, higher than in the groups discussed above, but still, it was not statistically significant. The maximum fluorescence point did not change after antihypertensive therapy, while the signal of minimal fluorescence increased. These changes were not statistically significant.

### 3.6. Metoprolol (Table 5)

A statistically significant change in NADH level was obtained only in the metoprolol group. Increases of the baseline level, points of the maximal and minimal fluorescence were observed compared to the first visit. The difference between baseline value fluorescence and minimal fluorescence, described by the Rmin parameter and indirectly with CImax, changed significantly after treatment.

**Figure 3:**
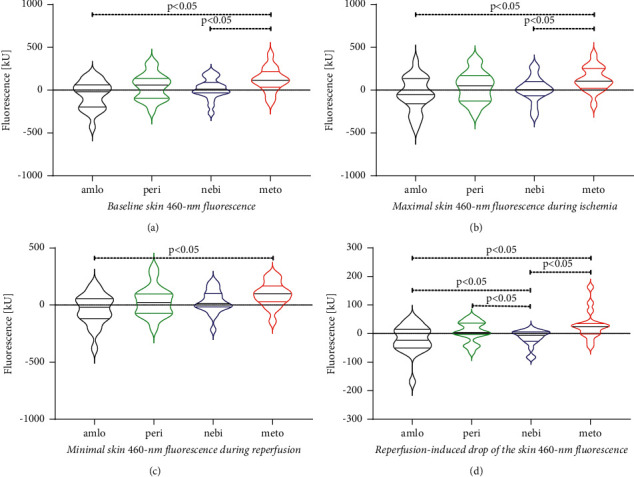
Violin plots with median (black bold lines) and values of the interquartile ranges (thin lines in the colours of the respective violin shapes) for the FMSF parameters, which differed significantly between visit 1 and visit 2. Results for patients randomised to once a day 5 mg of amlodipine are shown as black violins, to 5 mg of perindopril as green violins, to 5 mg of nebivolol as blue violins, and to twice a day 50 mg of metoprolol as red violins. In panel S, the between-visits' differences in the Bmean are presented, in panel B in the FImax, in panel C in the FRmin, and in panel D in the Rmin. For explanations of the FMSF parameters, refer to the main text in the methodology section and Figure 2.

The changes in fluorescence signal values between visits according to different tissue blood supply states are presented in Figure 3.

## 4. Discussion

FMSF parameters did not differ between the control and hypertensive patients suggesting that this disease, at least in grade 1 without organ complications, does not affect the NADH metabolism in the skin. Six-week treatment with amlodipine, perindopril, and nebivolol appears not to affect the FMSF curve. However, this curve increased after the treatment with metoprolol. To date, no studies have compared the effects of amlodipine, perindopril, nebivolol, and metoprolol on the NADH metabolism prospectively.

The decrease in blood pressure due to the applied therapy and the heart rate in the case of drugs from the *β*-blocker group was similar to the previously published ones [18–20]. Notably, the hypotensive effect between drugs was the same, so the changes in NADH signal were not associated with a decrease in blood pressure or a decrease in heart rate in the case of *β*-blockers. Therefore, we assume that metoprolol caused the FMSF parameters directly or indirectly rather than its hypotensive or negative chronotropic effects.

The potential mechanisms underlying the influence of the hypotensive drugs, particularly metoprolol, on the change FMSF curve may be complex. The parameters of the FMSF method mainly describe the function of microcirculation, like other methods using temporary ischemia. However, due to the assessment of NADH fluorescence, a particle of mainly mitochondrial origin also reflects the metabolic state of cells [3]. The FMSF method examines only a fragment of the forearm skin, but this model provides a picture of the state of the body's microcirculation in general [10].

The first mechanism may be related to metoprolol-induced changes in oxygen delivery to skin cells and mitochondria, which depend on blood flow and skin perfusion. However, the direct effect of metoprolol on mitochondria cannot be excluded.

Metoprolol is a lipophilic *β*-blocker, selective to *β*-1 adrenergic receptors. This drug has a weak membrane-stabilizing action (MSA) but without intrinsic sympathomimetic action (ISA) and alfa-blocking action [21]. By comparison, nebivolol—another lipophilic *β*-blocker—is more cardioselective than metoprolol, also without ISA and MSA but with significant vasodilator properties through direct stimulation of nitric oxide synthesis [22, 23].

There are scientific indications that metoprolol damages the microcirculation function or at least does not improve. Parameters indicating small vessels' condition are much worse than the effect of other *β*–blockers [18].

Velasco et al. showed that metoprolol reduces the microvascular response to exercise in people with uncomplicated hypertension. A similar effect was not observed with nebivolol [18]. Metoprolol, but not nebivolol, hindered the recruitment of new microcirculation vessels. Metoprolol significantly attenuated the increase in microvascular blood volume during handgrip but without alterations in the microvascular flow velocity, implying impaired vasodilation at the precapillary arterioles. Such an action of metoprolol might limit blood supply to peripheral tissues, including the skin, and consequently, decrease oxygen availability.

Veld et al. compared various effects of *β*-blockers in the short- and long-term. Acute infusion caused a small drop in blood pressure and increased vascular resistance proportional to the degree of cardiopression expressed by cardiac output. That suggested increased vasoconstrictor nerve activity via baroreflex protected against a sudden drop in blood pressure. Long-term supply was associated with a greater drop in blood pressure due to decreased vascular resistance, and it was based on partial agonist activity and possibly presynaptic beta-blockade [24]. In the case of metoprolol, vascular resistance increased in response to both short-term and long-term supply, but to varying degrees—the effect was weaker in the case of long-term supply [25–27].

Metoprolol also showed no beneficial effect on aortic endothelial function in diabetic hypertensive rats compared to nebivolol, which positively influenced the nitric oxide (NO) to peroxynitrite (ONOO^−^) ratio [28]. Hence, in hypertensive patients, metoprolol does not protect blood vessels and their endothelium from the adverse effects resulting from the disease.

In another study, in nephrectomised rats, Gschwend et al. showed that metoprolol, in contrast to nebivolol, strongly decreased endothelium-dependent endothelium-derived hyperpolarizing factor-mediated dilation. It is another premise for impaired microcirculation in people using metoprolol [29].

The action of metoprolol causes side effects such as muscle cramps [30] and cold hands and feet [31]. It suggests a reduction in the availability of oxygen and a reduction in blood supply to the tissues.

The direct influence of metoprolol on arterial resistance might explain reduced skin blood flow and oxygen delivery in HA patients treated with this agent and, probably, skin NADH accumulation observed in our study.

Interestingly, the effect of the drug on the quality of oxygen transport to cells might be the cause of the change in NADH levels. Previous studies have shown that the amount of NADH depended on the concentration of oxyhaemoglobin [4, 32]. Duman et al. showed that haemoglobin protein structure alters after binding with metoprolol [33]. Whether this could disrupt oxygen transport by haemoglobin and impact the NADH metabolism is uncertain.

Altogether, metoprolol may impair microcirculatory function and reduce cellular oxygen availability. The combination of these effects might explain an upward shift of the NADH fluorescence signal observed only in our patients treated with this drug.

Many *β*-blockers are potentially toxic to mitochondria [34]. *In vitro* studies have demonstrated that lipophilic *β*-blockers, such as metoprolol [35], inhibit the 3rd stage of cellular respiration in mitochondria [36]. The direct effect of *β*-blockers on mitochondria might explain chronic fatigue [36] in some patients, worsening sports performance in endurance athletes [37], respiratory failure in one child with mitochondrial disease [38], or worsening congenital myotonia in a person [39]. Sakurada et al. revealed that propranolol inhibited oxidative phosphorylation and oxidation of substrates combined with NAD+ and reduced the activity of enzymes and mitochondrial transporters [40]. In another study, carvedilol inhibited ATPase, stage-3-respiration, and complex I respiratory chain [34].

NADH exists as a free and protein-bound form, e.g., connected to the respiratory chain I complex, which cannot be distinguished by the FMSF technique [4]. NADH bound to proteins lasts longer [41], and this form mainly shows fluorescence [42]. The proportion of bound and free NADH is different according to tissue blood supply states [43]. If metoprolol interferes with the 3rd stage of cellular respiration, it may also affect the changes in NADH concentration bound by the complex I.

It appears that metoprolol directly impairs mitochondrial function and inhibits cellular respiration. Such effects might be responsible for increased NADH amount in skin cells demonstrated by an elevated run of the whole FMSF curve. The value of Rmin, describing the rate of oxidation of the accumulated NADH during an early phase of postischemic reperfusion, was higher after the six-week treatment with metoprolol. Perhaps during this treatment, mitochondria also changed their structure in response to some undetermined effects of metoprolol, like less than optimal oxygen supply. Depending on the tissue blood and oxygen supply, mitochondria may transform through their fusion and cleavage [42, 44] and reorganise respiratory complexes to optimise the electron flux [45]—such changes were observed even after a short duration of ischemia and reperfusion [44]. Pouli et al. also described that ischemia-induced mitochondrial dysfunction persists during reperfusion, despite the NADH signal returning to the basal line [42].

The limitations of the presented study are as follows. First, the studied group was relatively small. However, all patients were previously untreated and agreed to undergo time-consuming diagnosis to exclude secondary causes of hypertension. Secondly, mitochondrial function was assessed with the indirect method of FMSF measuring skin fluorescence at light length quite specific for NADH.

There are no data on the effects of the hypotensive drugs on NADH, and our results are the first to describe these phenomena. However, this issue needs further research, and this study has put a background for this.

## 5. Conclusion

All in all, this study indicates that newly diagnosed arterial hypertension without organ complications does not affect FMSF parameters compared to healthy people. However, the applied antihypertensive may modify the FMSF curve. Whereas amlodipine, perindopril, and nebivolol appear to be neutral, it seems that metoprolol increases the skin NADH content and its metabolism during short-lasting ischemia followed by reperfusion.

## Figures and Tables

**Table 1:** Characteristics of the study group, means (±SD). *N*—number of women and %—the percentage of women in the group.

Parameter	All patients	Amlodipine	Perindopril	Nebivolol	Metoprolol	p (ANOVA test)
Female (*N*/%)	29/38	7/33	8/42	7/35	7/44	0.262
Age (year)	40.0 (±12.2)	43.2 (±15.0)	38.8 (±10.9)	38.9 (±8.9)	38.8 (±13.5)	0.582
BMI (kg/m^2^)	29.1 (±5.2)	29.2 (±6.4)	28.7 (±4.0)	29.9 (±5.5)	28.2 (±4.7)	0.783

**Table 2:** Summary of continuous measures for hypertensive patients assigned to amlodipine at 5 mg QD. *P* values of the paired analysis of continuous data with a nonparametric Wilcoxon test for comparison of results before and after the six-week treatment.

Parameter	Median v1 (Q1–Q3)	Median v2 (Q1–Q3)	*P*
DTHR (beats/min)	81.61 (75.95–87.46)	81.90 (74.72–87.54)	0.2046
DTSBP (mmHg)	145.22 (141.85–154.36)	140.98 (136.55–146.90)	0.0106
DTDBP (mmHg)	87.92 (83.59–92.51)	83.37 (79.43–88.27)	0.0024
NTHR (beats/min)	61.54 (56.36–68.58)	65.86 (60.64–70.33)	0.4979
NTSBP (mmHg)	121.53 (113.81–130.17)	117.00 (110.63–120.33)	0.0143
NTDBP (mmHg)	71.43 (66.00–76.71)	68.88 (66.47–72.00)	0.0987
24-hHR (beats/min)	78.01 (72.81–85.27)	78.09 (72.46–84.27)	0.3945
24-hSBP (mmHg)	141.18 (138.24–148.63)	135.78 (133.58–142.01)	0.0087
24-hDBP (mmHg)	83.44 (80.70–88.84)	80.08 (77.69–85.35)	0.0046
Bmean (kFU)	698.76 (612.07–980.10)	650.87 (521.34–947.29)	0.1592
FImax (kFU)	764.51 (657.05–1020.35)	685.47 (591.92–1074.57)	0.4549
FRmin (kFU)	583.94 (512.90–837.28)	545.06 (453.11–756.72)	0.2891
Imax (kFU)	46.86 (29.45–85.08)	64.80 (32.17–88.77)	0.6639
Rmin (kFU)	136.79 (73.63–157.55)	105.81 (73.58–141.26)	0.0918
IRampl (kFU)	180.57 (106.74–222.38)	185.74 (118.52–210.54)	0.4761
CImax	0.32 (0.21–0.40)	0.38 (0.31–0.44)	0.2172

Bmean, mean fluorescence recorded before ischemia as the baseline value; FImax, the maximal fluorescence above the baseline during ischemia; FRmin, the minimal fluorescence below the baseline during reperfusion; Imax, the difference between FImax and Bmean; IRampl, the difference between FImax and FRmin; Rmin, the difference between Bmean and FRmin; CImax, Imax/IRampl rate; 24-hSBP, 24-hour systolic blood pressure; 24-hDBP, 24-hour diastolic blood pressure; 24-hPR, 24-hour pulse rate; DTSBP, day time systolic blood pressure; DTDBP, day time diastolic blood pressure; DTPR, day time pulse rate; NTSBP, night time systolic blood pressure; NTDBP, night time diastolic blood pressure; and NTPR, night time pulse rate.

**Table 3:** Summary of continuous measures for hypertensive patients assigned to perindopril at 5 mg QD. *P* values of the paired analysis of continuous data with a nonparametric Wilcoxon test for comparison of results before and after the six-week treatment.

Parameter	Median v1 (Q1–Q3)	Median v2 (Q1–Q3)	*P*
DTHR (beats/min)	78.85 (74.20–87.28)	81.63 (78.03–84.99)	0.5678
DTSBP (mmHg)	147.73 (141.83–156.44)	135.93 (133.37–139.86)	<0.0001
DTDBP (mmHg)	87.76 (82.28–91.98)	82.03 (78.62–83.93)	0.0002
NTHR (beats/min)	66.69 (58.26–71.43)	64.07 (58.15–68.93)	0.1956
NTSBP (mmHg)	121.00 (114.25–135.41)	112.80 (106.18–124.07)	0.0062
NTDBP (mmHg)	70.77 (63.30–78.86)	64.10 (60.83–67.68)	0.0053
24-hHR (beats/min)	75.93 (71.74–84.13)	75.95 (74.88–82.38)	0.9217
24-hSBP (mmHg)	142.14 (136.85–149.74)	130.08 (126.76–135.78)	<0.0001
24-hDBP (mmHg)	83.15 (78.51–89.06)	78.08 (73.76–80.81)	0.0001
Bmean (kFU)	573.97 (448.95–709.54)	603.27 (492.43–932.26)	0.3321
FImax (kFU)	601.06 (499.50–778.15)	646.14 (524.99–1034.60)	0.418
FRmin (kFU)	487.64 (375.46–600.34)	478.53 (409.05–782.31)	0.3736
Imax (kFU)	50.03 (30.78–79.26)	48.44 (32.79–70.67)	0.9843
Rmin (kFU)	90.97 (64.25–120.60)	99.04 (73.29–129.76)	0.4413
IRampl (kFU)	131.94 (113.13–183.81)	147.48 (111.40–192.99)	0.6507
CImax	0.35 (0.25–0.44)	0.34 (0.27–0.40)	0.4653

Bmean, mean fluorescence recorded before ischemia as the baseline value; FImax, the maximal fluorescence above the baseline during ischemia; FRmin, the minimal fluorescence below the baseline during reperfusion; Imax, the difference between FImax and Bmean; IRampl, the difference between FImax and FRmin; Rmin, the difference between Bmean and FRmin; CImax, Imax/IRampl rate; 24-hSBP, 24-hour systolic blood pressure; 24-hDBP, 24-hour diastolic blood pressure; 24-hPR, 24-hour pulse rate; DTSBP, day time systolic blood pressure; DTDBP, day time diastolic blood pressure; DTPR, day time pulse rate; NTSBP, night time systolic blood pressure; NTDBP, night time diastolic blood pressure; and NTPR, night time pulse rate.

**Table 4:** Summary of continuous measures for hypertensive patients assigned to nebivolol at 5 mg QD. *P* values of the paired analysis of continuous data with a nonparametric Wilcoxon test for comparison of results before and after the six-week treatment.

Parameter	Median v1 (Q1–Q3)	Median v2 (Q1–Q3)	*P*
DTHR (beats/min)	85.25 (80.67–91.59)	73.79 (66.34–84.34)	*<0.0001*
DTSBP (mmHg)	148.38 (142.81–150.98)	134.13 (129.21–139.13)	*0.0002*
DTDBP (mmHg)	88.76 (85.24–92.10)	79.25 (76.65–83.06)	*0.0004*
NTHR (beats/min)	67.87 (63.24–79.03)	59.08 (53.44–67.80)	*0.0003*
NTSBP (mmHg)	116.99 (108.50–127.35)	108.28 (103.93–118.27)	*0.0003*
NTDBP (mmHg)	69.36 (65.55–73.45)	64.19 (58.69–66.94)	*0.001*
24-hHR (beats/min)	82.97 (76.49–87.01)	71.00 (64.73–80.90)	*0.0001*
24-hSBP (mmHg)	142.23 (137.20–145.74)	128.89 (124.24–136.22)	*0.0001*
24-hDBP (mmHg)	84.20 (81.51–87.89)	76.40 (73.80–79.56)	*0.0002*
Bmean (kFU)	698.38 (480.04–790.76)	708.93 (479.29–869.10)	*0.498*
FImax (kFU)	743.52 (519.45–909.43)	745.37 (511.24–980.34)	*0.7285*
FRmin (kFU)	553.30 (404.35–624.36)	592.01 (416.14–725.93)	*0.2774*
Imax (kFU)	50.96 (17.29–89.98)	44.16 (16.28–80.50)	*0.8408*
Rmin (kFU)	132.77 (73.64–158.75)	115.55 (60.18–150.60)	*0.0696*
IRampl (kFU)	203.45 (98.85–235.99)	158.62 (90.14–223.12)	*0.165*
CImax	0.30 (0.22–0.39)	0.31 (0.24–0.37)	*1*

Bmean, mean fluorescence recorded before ischemia as the baseline value; FImax, the maximal fluorescence above the baseline during ischemia; FRmin, the minimal fluorescence below the baseline during reperfusion; Imax, the difference between FImax and Bmean; IRampl, the difference between FImax and FRmin; Rmin, the difference between Bmean and FRmin; CImax, Imax/IRampl rate; 24-hSBP, 24-hour systolic blood pressure; 24-hDBP, 24-hour diastolic blood pressure; 24-hPR, 24-hour pulse rate; DTSBP, day time systolic blood pressure; DTDBP, day time diastolic blood pressure; DTPR, day time pulse rate; NTSBP, night time systolic blood pressure; NTDBP, night time diastolic blood pressure; and NTPR, night time pulse rate.

**Table 5:** Summary of continuous measures for hypertensive patients assigned to metoprolol at 50 mg BID. *P* values of the paired analysis of continuous data with a nonparametric Wilcoxon test for comparison of results before and after the six-week treatment.

Parameter	Median v1 (Q1–Q3)	Median v2 (Q1–Q3)	*P*
DTHR (beats/min)	81.11 (77.02–89.21)	68.36 (65.93–73.73)	*<0.0001*
DTSBP (mmHg)	149.14 (143.49–156.49)	138.18 (128.65–144.28)	*0.001*
DTDBP (mmHg)	89.21 (84.26–92.83)	82.29 (74.88–84.93)	*0.0017*
NTHR (beats/min)	64.04 (60.76–67.59)	57.61 (53.02–63.46)	*0.0003*
NTSBP (mmHg)	130.76 (120.71–137.34)	114.65 (107.48–123.16)	*0.0003*
NTDBP (mmHg)	73.63 (68.52–81.62)	62.42 (58.17–69.15)	*0.0013*
24-hHR (beats/min)	77.74 (74.06–83.78)	67.13 (63.82–70.69)	*<0.0001*
24-hSBP (mmHg)	144.73 (141.09–150.52)	133.88 (125.23–138.88)	*0.0004*
24-hDBP (mmHg)	83.86 (81.57–90.38)	79.24 (71.47–81.98)	*0.0008*
Bmean (kFU)	698.78 (508.65–847.20)	841.20 (614.19–950.31)	*0.0034*
FImax (kFU)	762.31 (545.56–901.12)	900.81 (658.66–1046.42)	*0.0034*
FRmin (kFU)	578.17 (425.36–715.79)	674.15 (508.32–804.51)	*0.0052*
Imax (kFU)	43.59 (38.66–72.42)	53.08 (35.33–109.49)	*0.9799*
Rmin (kFU)	108.34 (70.17–143.78)	139.98 (103.53–168.55)	*0.0214*
IRampl (kFU)	149.88 (104.86–223.99)	188.66 (150.42–264.23)	*0.0182*
CImax	0.32 (0.29–0.38)	0.34 (0.23–0.41)	*0.2312*

Bmean, mean fluorescence recorded before ischemia as the baseline value; FImax, the maximal fluorescence above the baseline during ischemia; FRmin, the minimal fluorescence below the baseline during reperfusion; Imax, the difference between FImax and Bmean; IRampl, the difference between FImax and FRmin; Rmin, the difference between Bmean and FRmin; CImax, Imax/IRampl rate; 24-hSBP, 24-hour systolic blood pressure; 24-hDBP, 24-hour diastolic blood pressure; 24-hPR, 24-hour pulse rate; DTSBP, day time systolic blood pressure; DTDBP, day time diastolic blood pressure; DTPR, day time pulse rate; NTSBP, night time systolic blood pressure; NTDBP, night time diastolic blood pressure; and NTPR, night time pulse rate.

## Data Availability

The data are available to the interested parties per request to the corresponding author.
